# An evaluation protocol of the GreenConnect Dementia Respite Project: An innovative respite to improve the quality of life of the people living with dementia and reduce carer burden

**DOI:** 10.1002/alz70858_098478

**Published:** 2025-12-24

**Authors:** Shahinoor Akter, Sean MacDermott, Irene Darmadi Blackberry, Dan Douglass, Gerard José, Anthony Couroupis, Jim Boyer, Tshepo Rasekaba

**Affiliations:** ^1^ John Richards Centre for Rural Ageing Research, La Trobe Rural Health School, La Trobe University, Albury‐Wodonga, VIC, Australia; ^2^ Care Economy Research Institute, La Trobe University, Albury‐Wodonga, VIC, Australia; ^3^ La Trobe University, Wodonga, VIC, Australia; ^4^ Heathcote Health, Heathcote, VIC, Australia; ^5^ Rural Care Australia, Mildura, VIC, Australia; ^6^ Princes Court Ltd, Mildura, VIC, Australia; ^7^ Heathcote Dementia Alliance, Heathcote, VIC, Australia

## Abstract

**Background:**

Innovative respite services are crucial for enhancing the quality of life (QoL) of people living with dementia and carer dyads, particularly in resource‐poor rural areas. Evidence suggests that respite care based on Green Care principles – interventions involving nature elements ‐ can improve overall health of people living with dementia and their carers, while also promoting active community engagement. However, systematic research on the feasibility and impact of Green Care for dementia respite is limited in Australia. This protocol outlines a mixed‐methods study evaluating the implementation and outcomes of the GreenConnect Dementia Respite Project in regional Victoria, Australia. The Project aims to improve the QoL for people living with dementia, reduce caregiver burden and foster positive respite experiences in rural areas.

**Methods:**

The GreenConnect Dementia Respite Project has three phases: 1) Codesign phase; 2) Implementation phase and 3) Evaluation phase. A comprehensive mixed‐methods evaluation guided by the RE‐AIM (Reach, Effectiveness, Adoption, Implementation, Maintenance) framework will be used to assess the Project's impact on target population. Quantitative data on QoL (DEMQOL) and carer burden (Zarit Burden Interview) will be collected at baseline, 3 months, and 12 months post‐implementation. A satisfaction survey will be conducted after each tailored nature‐based activity. To capture participants and staff experiences, semi‐structured interviews with dyads and focus group discussions with project staff will be conducted at 12 months.

**Results:**

In the codesign phase, one consultation workshop with project consortium partners and steering committee members and two focus groups with carers of people living with dementia were held between November 2023 and January 2024 to inform implementation design and model of care. The implementation phase commenced in April 2024 and will continue until June 2026, with an anticipation of engaging up to 250 people with dementia and carers in nature‐based activities throughout the Project's lifetime. We present a protocol for evaluating the feasibility and efficacy of innovative nature‐based dementia‐specific respite care to improve QoL for individuals living with dementia and reducing caregiving burden.

**Conclusion:**

The project findings will provide evidence for future scalability and supporting the integration of nature‐based approaches into respite care services.

